# Homotherapy for Heteropathy: A Molecular Mechanism of Poria Sini Decoction for Treatment of Liver Cancer and Chronic Heart Failure

**DOI:** 10.1155/2024/9958258

**Published:** 2024-04-29

**Authors:** Zhe Zhao, Huiying Yue, Xiaohua Cui

**Affiliations:** ^1^Department of Second Clinical Medicine, Shanxi Medical University, Taiyuan 030001, China; ^2^College of Basic Medical Sciences, Shanxi University of Chinese Medicine, Taiyuan 030001, China; ^3^Department Cellar Biology and Genetics, Basic Medical College, Shanxi Medical University, Taiyuan 030001, China

## Abstract

Poria sini decoction (PSD), a significant traditional Chinese herbal formula, is effective in liver cancer (LC) and chronic heart failure (CHF); however, little is known about its concurrent targeting mechanism. *Methods*. This study analyzed the potential molecular mechanism of PSD against the two distinct diseases using network pharmacology approaches, including multidatabase search, pharmacokinetic screening, network construction analysis, Gene Ontology (GO) enrichment analysis, Kyoto Encyclopedia of Genes and Genomes (KEGG) pathway analysis, and molecular docking to elaborate the active components, signaling pathways, and potential mechanisms of PSD in the treatment of both LC and CHF. *Results*. A total of 155 active components and 193 potential targets in PSD were identified. Bioinformatics analysis revealed that quercetin, isorhamnetin, and naringenin, etc. may be potential candidate agents. TNF, AKT1, and IL6, etc. could become potential therapeutic targets. TNF-*α*, NF-*κ*B, PI3K-AKT, and TRP signaling pathways might play an important role in PSD against LC and CHF. Molecular docking results showed that most screened active compounds could embed itself into target proteins with a high binding affinity, and the hydrogen bonds number ≥3 indicated a more stable conformation of the compounds and target proteins. Overall, quercetin and isorhamnetin were the main active components, and TNF and AKT1 were the primary targets for PSD treatment of LC and CHF. *Conclusions*. This study illustrated that quercetin contained in PSD played an important role in the treatment of LC and CHF by acting on the key gene of TP53 and downregulating the PI3K-AKT signaling pathway.

## 1. Introduction

Chronic heart failure (CHF) and liver cancer (LC) are serious threats to human health. CHF is a complex clinical syndrome that results from any structural or functional impairment of ventricular filling. The high mortality and readmission rates of CHF are increasing due to ageing population [1]. LC is an extraordinarily heterogeneous malignant disease. According to a report published by the International Agency for Research on Cancer, the survival rate of LC is only 20% at present, and its mortality rate has increased to 70%. LC is not easy to be detected for the hidden onset and rapid progression, and once confirmed, it is usually at an advanced stage and difficult to cure [2]. Generally, these two diseases have the common characteristics of complicated aetiology, diverse syndromes, and long course. So, it is urgent to take effective prevention and treatment measures to reduce the incidence of CHF and LC. Modern medicine displays short-term clinical efficacy; however, different degrees of side effects occur with the prolongation of the treatment, which will bring certain economic burden and psychological impact to patients. Traditional Chinese medicine has the unique advantages of overall regulation and less adverse reactions in the treatment of LC and CHF [3, 4].

Poria sini decoction (PSD) originated from treatise on exogenous febrile diseases, and it comprised the following 5 kinds of traditional Chinese medicines: *Poria cocos* (Fuling), Panax Ginseng C. A. Mey. (Renshen), *Zingiberis rhizoma* (Ganjiang), liquorice (Gancao), and Aconiti Lateralis Radix Praeparata (Fuzi). It is found that PSD has a clear effect on the treatment of LC or CHF, respectively, through sorting out a large number of previous relevant literature and experimental verification. Urotensin II is one of the most potent vasoconstriction peptides, and it takes part in the pathological process of CHF. It is reported that PSD could downregulate the plasma urotensin II, thus improving the clinical symptoms of CHF [5]. Clinical experimental studies have proved that PSD effectively improved the clinical left ventricular ejection fraction, cardiac systolic function, and blood circulation function [6, 7], whereas greatly reduced the occurrence of arrhythmia, nausea, and vomiting and other adverse reactions during the treatment of CHF [8]. It is also reported that PSD improved liver function and immunologic function of LC patients; therefore, the patient's life is improved and survival time is significantly prolonged [9]. Studies have shown that PSD can regulate the immune system by upregulating the expression of P53 protein in hepatocellular carcinoma tissue, thereby inducing apoptosis of LC cells [10, 11]. Tuckahoe in PSD is shown to be quite effective for LC by inhibiting the expression of NLRP3/caspase-1/GSDMD in the classical pyroptosis pathway [12].

There is a complex relationship between liver disease and heart failure in the clinic, thus LC and CHF are often coexisting. That is, LC often causes increased blood pressure in patients and increases the risk of a series of cardiovascular diseases [13]. Besides, CHF results in congestive liver damage and ischemic liver disease and even lead to a vicious cycle of aggravating LC-related damage [14]. Consequently, when treating CHF, if have LC, must treat it at the same time, otherwise ineradicable. Even without LC, it is especially important to prevent and limit LC. As mentioned above, PSD can effectively treat LC and CHF, respectively, through the efficacy coordination among various herbs. However, there are rare reports on the biological mechanism of PSD in the simultaneous treatment of both LC and CHF, and the “homotherapy for heteropathy” molecular mechanism of PSD is still obscure. This study aims to explore the mechanism of PSD in the simultaneous treatment of both LC and CHF through network pharmacology. The study will inspire more innovative studies and reference for further experimental research and clinical application on the modern research of TCM formulas.

## 2. Materials and Methods

### 2.1. Screening Compounds and Targets of PSD

The active compounds of PSD were obtained from TCMSP (Traditional Chinese Medicine Systems Pharmacology Database and Analysis Platform) and the compounds were screened out under the conditions of the components' oral bioavailability (OB) ≥30% and drug likeness (DL) ≥0.18. The targets of active ingredients were obtained from the TCMSP and Swiss Target Prediction databases. Then, all these obtained active compounds were synthesized to remove duplications. Finally, the targets were standardized in the UniProt database with status set as “reviewed” and species set as “human,” which were all standardized and normalized to ensure accuracy.

### 2.2. Screening Targets of LC and CHF

The potential targets of LC and CHF were obtained from OMIM, GeneCards, and DrugBank databases using the keyword “liver cancer” or “chronic cardiac failure.” The obtained data were combined separately, and the duplications were removed, which were also all standardized and normalized to ensure accuracy.

### 2.3. Common Target Protein-Protein-Interaction Network Construction

The related targets of LC, CHF, and PSD were matched and a Venn diagram was drawn to obtain the common targets of the active compounds of PSD for treating LC and CHF. To further identify the core regulatory targets, protein-protein-interaction (PPI) analysis was performed by submitting overlapping targets to the STRING database. The species was limited to “Homo sapiens,” the medium required interaction score was set to 0.4, and the independent target protein nodes were hidden. In the end, the PPI results were exported from STRING and imported into Cytoscape 3.9.0 for PPI network construction and visualization.

### 2.4. “P-C-T-D” Network Construction

Intersections of PSD targets and disease-related targets were regarded as potential targets of PSD for the treatment of LC and CHF. The corresponding chemical compounds of the intersecting targets were thought to be possible therapeutic components that affected LC and CHF. The “PSD-compounds-targets-diseases” (P-C-T-D) network was constructed to clarify the relationship between active compounds from PSD and potential targets. This network was constructed and visualized using Cytoscape 3.9.0 software.

### 2.5. GO and KEGG Enrichment Analysis

Gene Ontology (GO) and Kyoto Encyclopedia of Genes and Genomes (KEGG) pathway enrichment analysis of drug-disease common targets were conducted through Metascape platform. The GO analysis included biological progress (BP), cellular component (CC), and molecular functions (MF) and then the top 20 results were visualized according to the ranking of *P* values.

### 2.6. Molecular Docking Verification

Molecular docking was performed between the active ingredients and the core targets obtained from PPI network topology analysis. The PDB formats of proteins were derived from the RCSB Protein Data Bank database (PDB), and SDF formats of ligands were obtained from the PubChem database. The 3D structure transformation of compounds and the format transformation of compounds and target proteins were completed by AutoDock Tools and OpenBabel software, respectively. Finally, Autodock 4.2 software was used for molecular docking and PyMOL was used for visualization.

## 3. Results

### 3.1. Active Compounds of PSD

A total of 155 candidate active ingredients were screened through TCMSP database, including 15 from *Poria cocos*, 22 from Panax Ginseng C. A. Mey., 5 from *Zingiberis rhizoma*, 92 from liquorice, and 21 from Aconiti Lateralis Radix Praeparata, respectively (the data were not shown). Among them, some active ingredients were shared by different drugs, for example, kaempferol was shared by Panax Ginseng C. A. Mey. and *liquorice*, *β*-sitosterol was shared by Panax Ginseng C. A. Mey. and *Zingiberis rhizoma*, and sitosterol was shared by *Zingiberis rhizoma*, liquorice, and Aconiti Lateralis Radix Praeparata. It is reported that kaempferol inhibited the proliferation, migration, and invasion of liver cancer HepG2 cells by the downregulation of microRNA-21 [15]. Sitosterol and *β*-sitosterol both belong to sitosterols, and they exert an anticancer effect in liver cancer cells by activating the caspase-3 pathway associated with apoptosis cell death [16].

### 3.2. Target Prediction of PSD in the Treatment of CHF and LC

**Figure 1:**
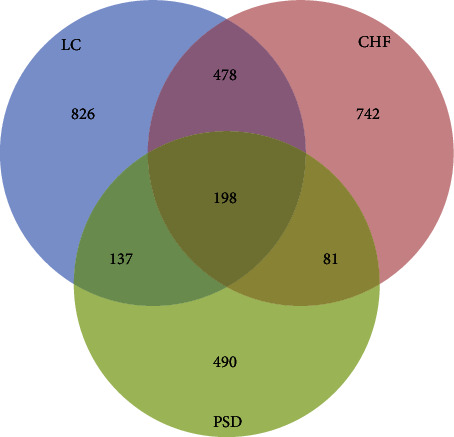
Targets matching among PSD (poria sini decoction), LC (liver cancer), and CHF (chronic heart failure).

After combining the three databases and deleting repeated targets, a total of 1641 targets for LC and 1499 targets for CHF were collected. Meanwhile, 906 active ingredient targets of PSD were obtained by integrating the targets obtained from the SWISS database and TCMSP database. Potential targets of PSD were matched with those of LC and CHF; then, a Venn diagram was constructed to visualize the data (as shown in Figure 1). The result showed that the intersections composing of 193 targets might be the potential drug target for the treatment of LC and CHF.

### 3.3. PPI Network and Analysis

**Figure 2:**
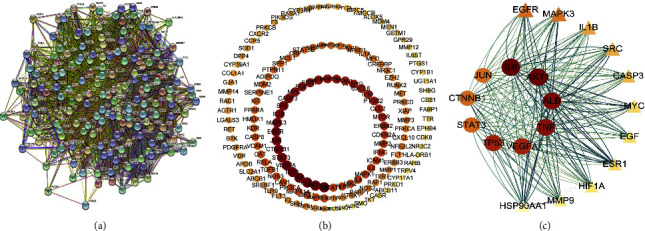
Protein-protein-interaction (PPI) network of core targets. (a) PPI networks of components of PSD (poria sini decoction) for the treatment of LC (liver cancer) and CHF (chronic heart failure) (each node represents the relevant targets, and edges stand for protein-protein associations). (b) PPI network imported from STRING database to Cytoscape 3.8.0. (c) Core targets of overlapping targets (red indicates a higher degree and pink represents a lower degree).

To explore the mechanism of PSD in the treatment of LC and CHF, the 193 target proteins of PSD against LC and CHF were imported into the STRING database for PPI analysis. As shown in Figure 2(a), each node represents relevant targets and edges stand for protein-protein associations. The network comprises 187 protein targets (nodes); they then interact with each other closely via 5316 edges. The PPI network was visualized by Cytoscape software to screen out the core targets, and median degree centrality (DC) values of the whole network was calculated to be 50. The targets with degree centrality (DC) values greater than 1 times the median or 2 times the median were extracted. As was shown in Figure 2(b), the outermost circle is all the 187 targets, the middle circle is the target whose DC value is more than 1 median, and the innermost circle is the target whose DC value is more than 2 median, which is the core targets of this study. Finally, aiming to analyze the relationship between the core targets (from the innermost circle of Figure 2(b)), the core targets were sorted in descending order according to its degree values by Cytohubba plugin (Figure 2(c)). The results suggested that IL6, AKT1, and TNF, etc. were the most effective core targets, and they interacted with each other.

### 3.4. “P-C-T-D” Network and Analysis

**Figure 3:**
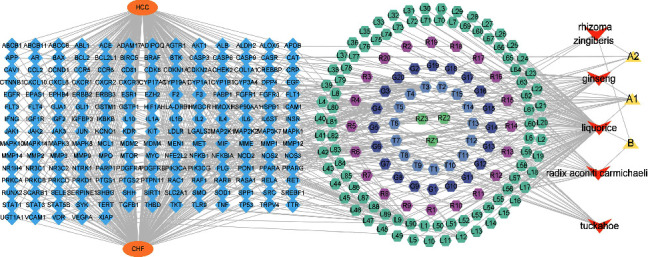
“Drug-component-target-disease (P-C-T-D)” network of PSD (poria sini decoction) against LC (liver cancer) and CHF (chronic heart failure) (inverted triangles are for traditional Chinese medicine; hexagons are different compound; rhombus are target; ellipses are names of disease. T: *Poria cocos*; G: Panax Ginseng *C*. A. Mey; RZ: *Zingiberis rhizoma*; L: liquorice; R: Aconiti Lateralis Radix Praeparata; A1: common components of Panax Ginseng C. A. Mey and liquorice; A2: common components of Panax Ginseng C. A. Mey and *Zingiberis rhizoma*; B: common components of *Zingiberis rhizoma*, liquorice, and Aconiti Lateralis Radix Praeparata.

**Figure 4:**
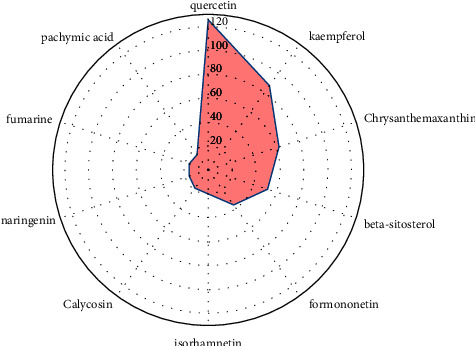
Degree values of key active ingredients.

The “drug-component-target-disease” network is shown in Figure 3, which contained a total of 344 nodes (193 intersection target genes, 155 active components, 2 diseases, and 5 active ingredients) and 1060 interacting edges. Each edge represented the relationship between the drug, the active ingredient and the disease. Among the 5 components of PSD, liquorice had the largest proportion of active ingredients and the most connected drug genes, indicating that liquorice had the strongest effect in the treatment of both of LC and CHF. In the order of connectivity value, quercetin, kaempferol, and chrysanthemaxanthin were the most active ingredients, suggesting that these ingredients might have better therapeutic effect. Among the 193 intersecting genes, IL6 had the highest degree of connection with the active ingredient, followed by AKT1, ALB, TNF, and TP53, etc. The higher the degree of connection, the stronger the target effect. The top 10 key active ingredients of PSD in the treatment of both LC and CHF ordered according to degree value is shown in Figure 4. It was seen from Figure 4 that the value of quercetin was the highest, followed by kaempferol. The higher the degree value, the larger the proportion, indicating that its activity was the strongest.

### 3.5. GO and KEGG Pathway Enrichment Analysis

**Figure 5:**
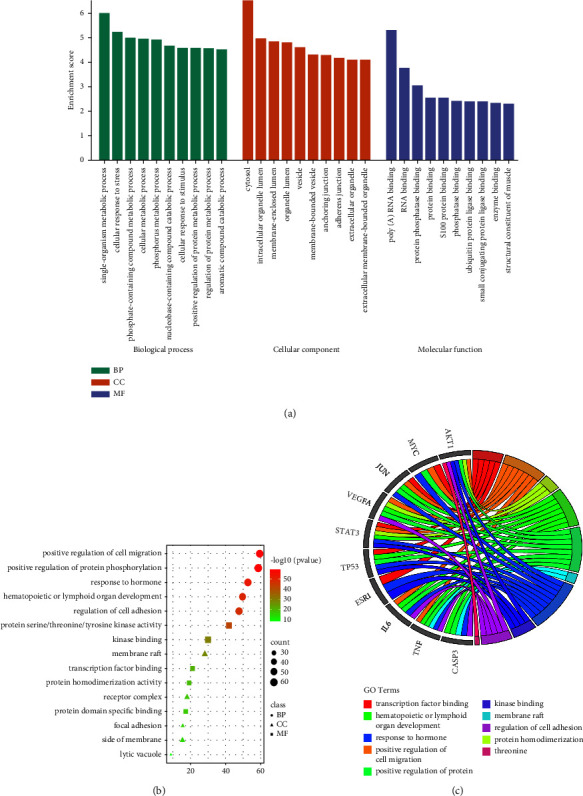
Gene Ontology (GO)-based enrichment analysis, as shown in bar chart (a), circle diagram (b), and bubble graph (c).

**Figure 6:**
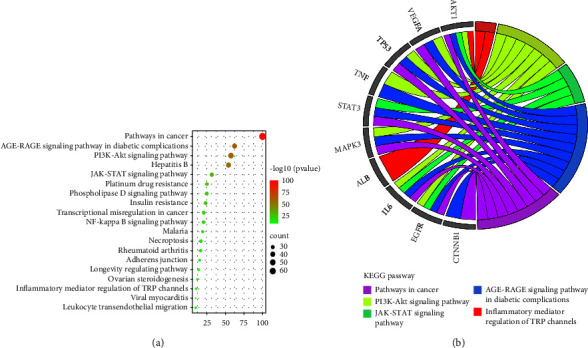
Kyoto Encyclopedia of Genes and Genomes (KEGG)-based enrichment analysis as shown circle diagram (a) and bubble graph (b).

The biological functional processes and molecular pathways of the crucial targets from PSD for treating LC and CHF were enriched and characterized, respectively. A total of 220 biological processes (BPs) were obtained, 188 cellular component (CC), and 188 molecular function (MF). The top items were selected based on the *P* value for visual analysis (Figures 5(a)–5(c)). Results showed that PSD treatment of LC and CHF mainly involves cell migration such as positive regulation of protein phosphorylation, regulation of cell adhesion, and kinase binding, etc. 207 signal pathways were obtained by enrichment analysis of the KEGG signal pathway. According to the *P* value, the top 20 items were screened out for visual analysis (Figures 6(a) and 6(b)). Moreover, Figure 6 showed that PSD treatment of LC and CHF might be mainly related to AGE-RAGE, PI3K-Akt, JAK-STAT, and other signaling pathways.

### 3.6. Molecular Docking Verification

**Figure 7:**
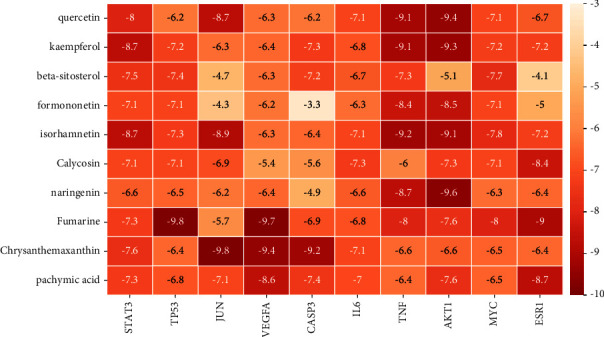
Docking results of target protein and active compound.

**Figure 8:**
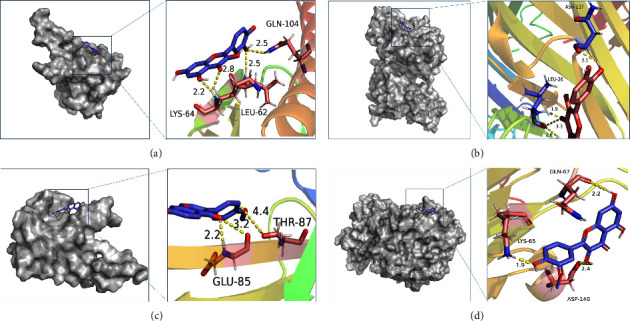
Molecular docking diagram of chemical composition to target: (a) AKT1-quercetin; (b) TNF-quercetin; (c) AKT1-formononetin; and (d) TNF-isorhamnetin.

According to Figure 7, the results showed that the binding energies of most compounds and ligands were less than −5.0 kcal·mol^−1^, indicating a good binding activity between them. The PDB protein database numbers of protein receptors are shown in Table 1. Among them, the binding energies of quercetin and kaempferol with STAT3, CASP3, IL6, and TNF were less than −7.0 kcal·mol^−1^, showing a strong binding activity. The ligand and receptor conjugates with stronger binding activity were visualized by PyMOL software, and the binding conformation with hydrogen bond number ≥3 was selected for display. As shown in Figure 8, quercetin forms 4 hydrogen bonds with AKT1 through amino acid residues LYS-64, LEU-62, and GLN-104 and forms 4 hydrogen bonds with TNF through amino acid residues LEU-26 and ASN-137. A higher number of hydrogen bonds indicate a more stable conformation of the ligand and acceptor. Furthermore, the abovementioned ligand compounds could be well embedded in the active pocket of the receptor target protein, suggesting that TP53 and JUN were the primary targets for PSD treatment of LC and CHF.

## 4. Discussion

### 4.1. Theoretical Explanation of “Homotherapy for Heteropathy” in PSD

As the essence of syndrome differentiation and treatment in traditional Chinese medicine, the theory of “homotherapy for heteropathy” also plays an important guiding role in the clinical diagnosis and treatment of traditional Chinese medicine [17]. The interpretation of “homotherapy for heteropathy” generally means that the same methods are adopted to cure different diseases showing similar symptoms during the development process, which fully reflects the advantages of traditional Chinese medicine in holistic and comprehensive treatment [18, 19]. Traditional Chinese medicine can treat different diseases simultaneously and prevent the occurrence of diseases by enhancing the body's defense and adaptability. Previous clinical studies have shown that PSD can treat a variety of diseases by regulating cardiac function, enhancing human immunity, and improving tumor microenvironment. Therefore, from the perspective of “homotherapy for heteropathy” as the starting point, the study combined network pharmacology and molecular docking technology to explore the common mechanism of PSD on LC and CHF and provided a basis for further clinical applications.

### 4.2. Main Active Components of PSD in the Treatment of LC and CHF

The “drug-component-target-disease” network of PSD in the treatment of LC and CHF obtained in the study showed that 10 compounds including quercetin, kaempferol, and isorhamnetin had high connectivity and degree values. These components might be important components of PSD in the treatment of LC and CHF, and most of them have been verified by clinical trials and related animal experiments. For example, (1) quercetin can not only reduce the incidence of CHF by protecting cardiomyocytes under inflammatory conditions but also enhance inhibitory effect on the growth of LC cells by binding 5-FU [20, 21]. (2) Kaempferol can not only inhibit the proliferation, migration, and invasion of liver cancer HepG2 cells by the downregulation of microRNA-21 but also decrease inflammation in Ang II-stimulated cardiac fibroblasts by modulating NF-kB pathways [15, 22]. Other research results are summarized in Table 2. These studies have confirmed that the active components of PSD play an important role in the treatment of LC and CHF.

### 4.3. Key Targets of PSD in the Treatment of LC and CHF

The results from PPI network and molecular docking showed that PSD had therapeutic effects on the abovementioned diseases through core targets such as STAT3, CASP3, IL6, TNF, and AKT1. Previous studies have shown that STAT3 inhibits LC cells migration and growth by controlling the progression of continuous hepatocyte death, inflammatory cell infiltration, and compensatory liver regeneration [29]. The IL-6-stat3 signaling pathway is blocked by the IL-6 monoclonal antibody and regulated by IL-6 miRNAs through multiple targets. These results suggested that abnormally proliferating IL-6 can inhibit the occurrence and development of LC by interfering tumor cell proliferation, migration, invasion, angiogenesis, and apoptosis [30]. TNF-*α* can effectively improve inflammatory factors or oxidative stress indicators, promote cardiomyocyte contraction, and decrease tumor activity indicators [31]. It is reported that TNF-*α* had a strong target benefit in LC as well as CHF when healing the CHF with l-carnitine combined with telmisartan [32]. Loss of function or death of AKT1 cardiomyocyte is one of the main factors in the development of CHF. Western blot phosphorylation assay ultimately proves that cytoplasmic Hsp60 is involved in the regulation of AKT1 activity in the progression of CHF [33]. TP53 mutation is the most common mutation in LC, and it affects the progression and prognosis of LC [34]. On the other hand, TP53 is involved in the pathogenesis of dilated cardiomyopathy [35]. It has been reported that dilated cardiomyopathy can progress to CHF [36].

Results of molecular docking further verified that the core targets such as STAT3, TP53, JUN, VEGFA, CASP3, IL6, and TNF had good binding ability with the key active components. The binding energy was frequently calculated to evaluate the affinity degree of ingredients with protein targets. The lower the binding energy, the more hydrogen bonds are formed, indicating a higher affinity of the ligand for the receptor. According to Figure 7, the binding energies of quercetin and kaempferol with STAT3, CASP3, IL6, and TNF were all less than −7.0 kcal·mol^−1^. Among them, in AKT1-quercetin interaction, the binding energy of quercetin and AKT1 was −9.4 kcal·mol^−1^; meanwhile, quercetin formed four hydrogen bonds with residues LYS-64, LEU-62, and GLN-104. Quercetin and AKT1 formed the largest number of hydrogen bonds and the lowest binding energy, indicating that these molecular docking results were the most stable structure. It is preliminarily verified that the binding of the main active components such as quercetin and isorhamnetin in PSD to the core target such as AKTI and TNF may be the key to its action. These results confirmed that PSD had a good therapeutic effect on LC and CHF.

### 4.4. Signaling Pathways of PSD in the Treatment of LC and CHF

The analysis of GO biological process and KEGG pathway of common targets showed that PSD treatment of LC and CHF mainly involved NF-*κ*B, PI3K-Akt, JAK-STAT, TRP signaling pathways and the biological process of protein phosphorylation. Studies have shown that PSD can activate the NF-*κ*B pathway by upregulating the expression of IL-6 and TNF-*α*. Meanwhile, PSD inhibits the inflammatory response through negative feedback, thereby alleviating myocardial depression in CHF patients [37]. PSD upregulates the expression of the proliferation inhibitory proteins P21 and P27 by regulating the expression of key molecules in the PI3K-Akt signaling pathway. This process can produce a significant tumor suppressor effect on H22 cells and enhance the anti-LC effect [38]. Animal experimental studies have shown that the JAK-STAT signaling pathway is activated by the reducing of Mir-195-5p or the upregulating of CXCR4, in turn, alleviated the damage of cardiac function in CHF. Besides, JAK-STAT signaling pathway participated in reducing the inflammatory factors contents, oxidative stress, and myocardial enzyme index [39]. It has also been reported that the JAK-STAT signaling pathway can use the high expression of microRNA-409 to delay the progression of LC [40]. For patients with CHF, sustained neurohumoral activation, pressure overload, or myocardial injury can cause cardiac pathological hypertrophic growth. TRP ion channels are critical for activation of cellular signaling pathways involved in adverse cardiac remodeling and CHF, which can also treat pain syndrome associated with LC. Therefore, TRP channels play a crucial role in the treatment of CHF and LC [41, 42]. Tyrosine phosphorylated proteins are mainly associated with biological processes such as adherens junctions, focal adhesions, endocytosis, and tight junctions. A variety of tyrosine phosphorylated proteins provide new therapeutic targets for the LC metastasis [43]. Clinical experiments show that the cardiac pathological changes and fibrosis are improved after Cap treatment, which significantly inhibits cardiac cell apoptosis in the process of CHF. Meanwhile, the expressions of phosphorylated Jak2 and Stat3 are significantly reduced, and the expression of Bcl-2 is increased [44]. A novel plaque-binding kinase inhibitor (CT-707) has therapeutic effects on LC. CT-707 can inhibit the proliferation of hepatoma cells and promote cell apoptosis. It is reported that focal adhesion can reduce the viability of hepatoma cells [45]. Therefore, it can be speculated that the main components of PSD plays a role in the treatment of LC and CHF.

## 5. Conclusion

In conclusion, the study performed network pharmacology and molecular docking analysis to elucidate that in the anti-LC and CHF effects of PSD, the most important bioactive compound was quercetin, TP53 was the most important target, and the PI3K-AKT signaling pathway was the most critical pathway. Although the network pharmacology research of TCM theory of “homotherapy for heteropathy” is still in the preliminary stage, with the continuous improvement of database information, future research will deepen people's understanding of TCM treatment of complex diseases. PSD can effectively treat LC and CHF through the efficacy coordination among various herbs. The idea of “homotherapy for heteropathy” is conducive to the timely discovery of new drugs and brings new hope for the treatment of LC and CHF.

## Figures and Tables

**Table 1:** Details of protein receptor's PDB ID for molecular docking.

Target protein	PDB ID
STAT3	3CWG
TP53	1KZY
JUN	5T01
VEGFA	4KZN
CASP3	2CJX
IL6	1ALU
TNF	5M2J
AKT1	2UZS
MYC	5I4Z
ESR1	1SJ0

**Table 2:** Experimental verification results of main chemical constituents.

Active ingredient	Disease	Treatment mechanism	Experiment model	Literature
Naringenin	LC	Naringenin induces growth inhibition, cell cycle arrest, and apoptosis in human hepatocellular carcinoma cells	Cell culture	[23]

Naringenin	CHF	Naringenin attenuates pressure overload-induced cardiac hypertrophy	Mice	[24]

Isorhamnetin	LC	Isorhamnetin induces ROS-dependent cycle arrest at G2/M phase and apoptosis in human hepatocarcinoma Hep3B cells	Cell experiment	[25]

Isorhamnetin	CHF	Isorhamnetin exerts a protective effect against myocardial injury through the attenuation of apoptosis	Cell culture	[26]
			Living organisms	

Calycosin	LC	Calycosin has a potential effect in inhibiting oncogene expression and increasing anticancer genes expression	Cell culture	[27]

Calycosin	CHF	Calycosin inhibited inflammation and fibrosis via activation of the PI3K-AKT pathway in postacute myocardial infarction rats	The rat	[28]

*Note*. LC: liver cancer; CHF: chronic heart failure.

## Data Availability

The data used to support the findings of this study are included within the article.
